# Associations between dietary patterns and stages of chronic kidney disease

**DOI:** 10.1186/s12882-022-02739-1

**Published:** 2022-03-22

**Authors:** Hsin-I. Lin, Hui-Ming Chen, Chien-Chin Hsu, Hung-Jung Lin, Jhi-Joung Wang, Shih-Feng Weng, Yuan Kao, Chien-Cheng Huang

**Affiliations:** 1 Department of Nutrition, Yuan’s General Hospital, Kaohsiung, Taiwan; 2 Department of Healthcare Administration and Medical Informatics, College of Health Sciences, Kaohsiung Medical University, Kaohsiung, Taiwan; 3 Center for Big Data Analytics and Statistics, Chang Gung Memorial Hospital, Linkou, Taiwan; 4 Department of Emergency Medicine, Chi Mei Medical Center, Tainan, Taiwan; 5 Department of Biotechnology, Southern Taiwan University of Science and Technology, Tainan, Taiwan; 6 Department of Emergency Medicine, Taipei Medical University, Taipei, Taiwan; 7 Department of Anesthesiology, Chi Mei Medical Center, Tainan, Taiwan; 8 Department of Anesthesiology, National Defense Medical Center, Taipei, Taiwan; 9 Department of Medical Research, Kaohsiung Medical University Hospital, Kaohsiung, Taiwan; 10 Center for Medical Informatics and Statistics, Office of R&D, Kaohsiung Medical University, Kaohsiung, Taiwan; 11 Center for Big Data Research, Kaohsiung Medical University, Kaohsiung, Taiwan; 12 Department of Medicine Science Industries, Chang Jung Christian University, Tainan, Taiwan; 13 Department of Emergency Medicine, Kaohsiung Medical University, Kaohsiung, Taiwan

**Keywords:** Chronic kidney disease, Stage, Dietary pattern, Nutrient intake

## Abstract

**Background:**

Studies have revealed that patients with chronic kidney disease (CKD) have dietary patterns different from those of the general population. However, no studies have compared the dietary patterns of between patients with early-stages (stages 1–3a) and late-stages (stages 3b–5) of CKD. Our objective was to investigate the associations between dietary patterns in early and late-stage CKD.

**Methods:**

We analyzed 4480 participants with CKD at various stages based on the data recorded between 2007 and 2016 from the database of the American National Health and Nutrition Examination Survey.

**Results:**

In total, 3683 and 797 participants had early and late-stage CKD, respectively. Through principal components analysis, the dietary intake dimension was reduced from 63 variables to 3 dietary patterns. We adopted logistic regression for analysis. The three dietary patterns are as follows: (1) saturated fatty acids and mono-unsaturated fatty acids (MUFA); (2) vitamins and minerals; and (3) cholesterols and polyunsaturated fatty acids (PUFA). These 3 patterns explained > 50% of dietary nutrient intake. Results indicated that among participants with dietary patterns 2 (vitamins and minerals) and 3 (cholesterols and PUFA), those with low intakes were more likely to have late-stage CKD. The odds ratios for patterns 2 and 3 were 1.74 (95% CI: 1.21–2.50) and 1.66 (95% CI: 1.13–2.43), respectively.

**Conclusions:**

This study revealed that intakes of vitamins and minerals and cholesterols and PUFA were associated with the stages of CKD.

**Supplementary Information:**

The online version contains supplementary material available at 10.1186/s12882-022-02739-1.

## Background

Changes in lifestyles and dietary habits, smoking, obesity, and aging as well as diabetes, hypertension, and hypercholesterolemia have attributed to increases in the prevalence rate of kidney diseases annually [1]. Approximately 15% of United States adults have chronic kidney disease (CKD) [1]. The kidneys of patients with CKD are partially impaired and are unable to metabolize excessive nutrients and toxins from the body. Thus, these patients are prone to hyperphosphatemia and secondary hyperparathyroidism. Cardiovascular diseases such as arterial stiffness and myocardial infarction can be caused by hyperphosphatemia, thus increasing the risk of death among these patients [2, 3].

Macronutrients are the elements, including carbohydrates, protein, and fat that our body need to grow and function normally [4]. Micronutrients are the elements that our body need in small amount such as vitamins and minerals [4]. Studies have indicated that enough intake of micronutrients can decrease the risk of CKD. In addition, reducing protein intake can lower the production of blood urea nitrogen and thus delay the course of CKD [5–7]. Van Westing et al. conducted a meta-analysis of 21 prospective cohort studies and found that healthy dietary habits (including consumption of vegetables, coffee, and dairy products) reduced the risk of CKD [8]. By contrast, unhealthy diets (including consumption of red meat, processed meat, and sweetened beverages) may promote kidney function loss [8]. A study that interviewed 211 patients with CKD revealed a direct association between high-fat dietary patterns and the progression of CKD [9]. Kidney function may deteriorate if the intake of vitamins, minerals, protein, and fat cannot be moderated among patients with CKD. Thus, the dietary intake conditions of patients with CKD should be evaluated with caution.

Diets include a complex variety of nutrients; people with regular diets are unlikely to ingest only a single nutrient. However, nutrition guides for patients with CKD mainly provide suggestions targeting macronutrients (e.g., energy and protein) and individual micronutrients [5, 10]. Other guides discuss the protective effects of special dietary patterns for CKD (e.g., Dietary Approaches to Stop Hypertension [DASH] and the Mediterranean diet) [11–13]. Few studies discussed the influences of the overall dietary intake on CKD [14, 15]. In addition, most studies on the associations between CKD and diet have only analyzed patients with CKD or high-risk individuals, such as those with hypertension, diabetes, and obesity [16, 17]. No studies have compared the associations of diet on the early (stages 1 to 3a) and late-stages (stages 3b to 5) of CKD.

Dietary intake has potential influences on the occurrence and progression of CKD, and most studies have indicated that the dietary patterns of patients with CKD differ from those of the general population [15]. However, no studies have explored the association between nutrient intake and the risks of the various stages of CKD. Therefore, this study analyzed indicators related to kidney disease, disease risk factors, and dietary patterns. Differences in dietary intake between patients with early and late-stage CKD were discussed.

## Materials and methods

### Data source

This study employed the database of the National Health and Nutrition Examination Survey (NHANES) and selected data on 50,588 individuals that was collected between 2007 and 2016 as the study sample. The NHANES is a cross-sectional representative survey conducted biannually by the National Center for Health Statistics of the United States Centers for Disease Control and Prevention [18]. Demographic, dietary intake, and behavior data were collected through individual interviews, questionnaire surveys, physical examinations, blood samples, urine samples, and nutritional evaluations [18]. The aim of this survey is to evaluate the health and nutritional status of adults and children in the United States in order to examine disease prevalence rate and trends among the general public [18].

### Recruitment of participants with CKD

**Fig. 1:**
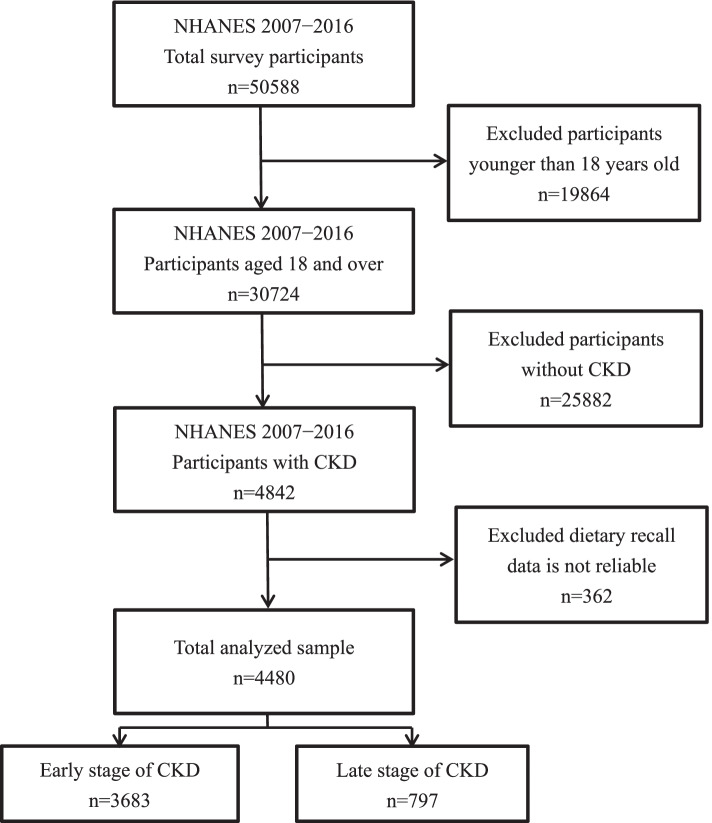
Flowchart of the screening process for the selection of eligible participants

The survey recorded samples of participants with various levels of CKD who were aged 18 years or older. Consequently, individuals aged younger than 18 years (*N* = 19 864), those without CKD (*N* = 25 882), and those with uncertain diagnosis were excluded. The Kidney Disease Improving Global Outcomes (KDIGO) recommends using estimated Glomerular filtration rates (eGFR) and albuminuria to determine the severity of CKD [19]. The course of CKD was divided into 5 stages (G1–G5) [19]. CKD-EPI was used to evaluate eGFR, and the severity of albuminuria was assessed based on the urine albumin creatinine ratio. The estimated Glomerular filtration rates was calculated by the equation eGFR = 141 × min (Scr/κ, 1)^α^ × max (Scr/κ, 1)^−1.209^ × 0.993^Age^ × 1.018 [if female] _ 1.159 [if black], where Scr is serum creatinine, κ is 0.7 for females and 0.9 for males, α is -0.329 for females and -0.411 for males. Afterward, 4 842 of the participants were diagnosis with CKD according by the classification standards of previous studies [20–22], and divided CKD stages into early-stages (G1 to G3a) and late-stages (G3b to G5). To ensure that the participants’ dietary memories were complete and reliable, incomplete answers to dietary memory questions and incomplete data were excluded. Finally, 4 480 participants were included in the sample data of this study and the early and late-stage CKD groups comprised 3 683 and 797 participants, respectively (Fig. 1).

### Basic information, related risk factors, and comorbidities

This study considered the following characteristics and risk factors related to CKD as independent variables: sex, age, race, body mass index (BMI), waist circumference, triglyceride, total cholesterol, high-density lipoprotein (HDL), triglyceride-to-HDL cholesterol ratio, apolipoprotein B, systolic blood pressure, diastolic blood pressure, fasting blood glucose, plasma insulin, homeostatic model assessment-insulin resistance (HOMA-IR), glycated hemoglobin (HbA1C), 2-h postprandial blood glucose, hypertension, diabetes, and metabolic syndrome. Blood pressure was recorded as the average value of 4 measurements, and the HOMA-IR was calculated as glucose level (mg/dL) × insulin level (μU/mL)/405. The diabetes and hypertension were assessed by asking participants whether they had been diagnosed with these diseases. Metabolic syndrome was assessed using the standards of the International Diabetes Federation and the American National Heart, Lung, and Blood Institution. If 3 or more of the following standard definitions were met, then metabolic syndrome was recorded: (1) male waist circumference ≥ 102 cm or female waist circumference ≥ 88 cm; (2) triglyceride level ≥ 150 mg/dL; (3) male HDL cholesterol level < 40 mg/dL or female HDL cholesterol level < 50 mg/dL; (4) systolic blood pressure ≥ 130 mmHg or diastolic blood pressure ≥ 85 mmHg or use of hypotensive drugs; (5) fasting plasma glucose level ≥ 100 mg/dL or use of hypoglycemic drugs [23]. We estimated eGFR by using the CKD-EPI formula.

### Diet review

The dietary memory data from the NHANES database recorded various food/beverage items and the quantities that participants consumed over a 24-h period starting from midnight of the day before the interview. To assist participants with accurately reporting the quantities of ingested food and drink, the records were documented with uniform measurement standards in the interviews. After the interviews, the energy and nutrients in the ingested food/beverage of participants were coded according to the U.S. Department of Agriculture’s Food and Nutrient Database for Dietary Studies (FNDDS), and the total intake quantities of nutrients were calculated.

### Ethical statement

The NHANES is a publicly available database and approved by the National Center for Health Statistics institutional review board. All methods were carried out in accordance with relevant guidelines and regulations. All participants provided written informed consents for their participation in the NHANES.

### Statistical methods

Numerous nutrition components are present in dietary intake; thus, factor analysis was required to generate representative dietary patterns for subsequent analysis. It is very difficult to investigate the effects of single foods on health outcomes because the consumption of foods is highly intercorrelated [24]. Therefore, epidemiologists specialized in nutrition are increasingly to focus on dietary patterns rather than on single foods or nutrients [24]. The principal component analysis (PCA), a posteriori (data-driven) approach, is a commonly used method for derive dietary patterns from habitual diet [24]. We adopted the PCA for factor sampling [24], and the varimax method was used for orthogonal rotation of the axis. This method enabled the generation of the principal components to represent dietary patterns. The Kaiser–Meyer–Olkin value of the dietary intake data was 0.90, and the results of Bartlett’s test of sphericity were significant (*P* < 0.001), indicating suitability for factor analysis. The results of PCA and scree plot analysis were integrated, and results with eigenvalue > 4 were retained as the factors for the final analysis. The extracted dietary patterns explained more than 50% of the dietary intake [25].

As part of factor analysis, we weighted the intakes of nutrients and calculated the participants’ scores for each dietary pattern. Higher scores indicate higher nutrient intakes in the dietary pattern. Because the dietary pattern scores did not belong to a normal distribution, the samples of different dietary patterns were assigned to 4 quartile groups with identical sizes. The dietary patterns less than the 25th percentile (Q1) were the low intake group, those from the 25th to 50th percentiles (Q2) were the low-to-middle intake group, those from the 50th to 75th percentiles (Q3) were the middle-to-high intake group, and those more than the 75th percentile (Q4) were the high intake group. This variable was also used in subsequent analysis, in which the high intake group was used as the reference group.

To discuss the associations between dietary patterns and the stages of CKD, this study employed descriptive statistics to analyze the characteristics of patients with early and late-stage CKD and the differences in CKD-related risk factors. In addition, multiple logistic regression was used to analyze the associations between dietary patterns and CKD stages. After performing factor analysis for the dietary intake data, the resulting factors were assessed in various models. Model 1 was only adjusted for age, sex, and race. Model 2 was adjusted for all the variables in model 1 as well as hypertension, HDL level, and triglyceride level. Model 3 was adjusted for all the variables in model 2 as well as BMI and diabetes. Moreover, the value of varimax rotated factor pattern of three factors by PCA was used to analyze by using general linear model to demonstrate the association between dietary patterns and CKD stages after adjusted all confounders. We used the SAS 9.4 software for analyses due to that NHANES data are saved in a SAS transport (.XPT) file. For every 2-year sample Mobile Examination Center (MEC) weights WTMEC2YR was used for all NHANES analyses. In addition, *P* < 0.05 (2-tailed) was considered as indicating statistical significance.

## Results

This study employed sample data from the NHANES database that was recorded between 2007 and 2016 and categorized CKD stages into early and late stages. The differences in the dietary patterns of the 2 groups were analyzed. After conditional exclusion, 4 480 samples were retained, which comprised of 3 683 and 797 samples which had early-stage CKD and late-stage CKD, respectively. Through eigenvalues and scree test in the PCA, the 63 diet record variables were reduced to 3 dietary patterns (Supplementary Table 1), namely saturated fatty acids and monounsaturated fatty acids (MUFAs) (pattern 1), vitamins and minerals (pattern 2), and cholesterols and polyunsaturated fatty acids (PUFAs) (pattern 3). These 3 dietary patterns explained > 50% of the dietary intake. In the scree test, the turning point of the dietary pattern is about point 3 (Supplementary Fig. 1), which indicates that 3 dietary patterns are most appropriate because components to the left of this point should be retained as significant [26].

Table 1 displays the overall characteristics and dietary statuses of the participants. In the dietary pattern analysis, female participants accounted for 57.93%, and the average age of the participants was 60 years. The average age of participants with late-stage CKD was higher than that of those with early-stage CKD (72.4 years vs. 57.8 years, *P* < 0.001). Most of the participants were Caucasian (70.01%), and African American participants accounted for approximately 12%. Participants with late-stage CKD were more likely to be non-Spanish Caucasian and non-Spanish African American than those with early-stage CKD; this trend was reversed for other races. The average waist circumference of the participants was 102.90 cm. Participants with late-stage CKD had significantly lower total cholesterol, HDL, and apolipoprotein B in their blood than those with early-stage CKD (*P* < 0.001). For HbA1c and 2-h glucose, participants with late-stage CKD had significantly higher values than those with early-stage CKD (*P* < 0.001). Among comorbidities, those with late-stage CKD had higher incidences of hypertension, diabetes, and metabolic syndrome than those with early-stage CKD (*P* < 0.001). More detail of each stage of CKD was showed in the Table 2.

**Table 1:** Demographic characters and related risk factors of persons based on late-stage CKD and early-stage of CKD

Characteristics		Overall Mean ± SD (%)	Late-stage of CKD (*n* = 797)	Early-stage of CKD (*n* = 3683)	*p*-value
Sex	Male (%)	42.07	35.97	43.17	< 0.001
	Female (%)	57.93	64.03	56.83	
Age (Years), [mean (95% CI)]		60.0(59.5–60.5)	72.4(71.7–73.2)	57.8(57.2–58.3)	< 0.001
Race/Ethnicity	Mexican American (%)	7.24	4.58	7.71	< 0.001
	Other Hispanic (%)	4.72	3.48	4.94	
	White (non-Hispanic) (%)	70.01	73.86	69.32	
	Non-Hispanic Black (%)	12.04	13.27	11.81	
	Other (%)	5.99	4.80	6.21	
BMI (kg/m^2^)		29.94 ± 0.11	30.23 ± 0.27	29.89 ± 0.12	0.264
Waist circumference (cm)		102.90 ± 0.27	104.60 ± 0.60	102.7 ± 0.30	0.004
Triglycerides (mg/dL)		141.50 ± 2.57	143.30 ± 5.19	141.10 ± 2.89	0.717
Total cholesterol (mg/dL)		192.60 ± 0.69	181.4 ± 1.59	194.6 ± 0.76	< 0.001
High density lipoprotein		52.92 ± 0.27	50.74 ± 0.57	53.31 ± 0.30	< 0.001
Serum triglycerides/HDL cholesterol ratio		3.28 ± 0.10	3.25 ± 0.16	3.28 ± 0.11	0.885
Apolipoprotein (B) (mg/dL)		92.20 ± 0.61	88.98 ± 1.59	92.74 ± 0.66	0.029
Systolic blood pressure (mmHg)		130.80 ± 0.33	134.00 ± 0.88	130.30 ± 0.36	< 0.001
Diastolic blood pressure (mmHg)		68.15 ± 0.23	61.06 ± 0.59	69.42 ± 0.24	< 0.001
Fasting blood glucose (mg/dL)		120.90 ± 1.12	121.50 ± 2.72	120.80 ± 1.22	0.805
Plasma Insulin (uU/mL)		15.68 ± 0.60	16.53 ± 2.47	15.54 ± 0.55	0.695
HOMA-IR		5.28 ± 0.26	5.73 ± 1.02	5.20 ± 0.24	0.615
HbA1c (%)		6.14 ± 0.02	6.28 ± 0.04	6.12 ± 0.02	< 0.001
2-h blood glucose (mg/dL)		138.6 ± 1.83	154.9 ± 4.81	136.6 ± 1.96	< 0.001
Hypertension (%)		58.07	83.49	53.48	< 0.001
Diabetes (%)		25.16	39.54	22.56	< 0.001
Metabolic syndrome (%)		39.14	52.32	36.76	< 0.001
Dietary pattern 1 [saturated fatty acids & MUFA] (%)					
High intake		27.68	24.13	28.32	< 0.001
Middle-to-high intake		26.26	24.08	26.65	
Low-to-middle intake		23.67	26.70	23.12	
Low intake		22.39	25.10	21.90	
Dietary pattern 2 [vitamins & minerals] (%)					
High intake		27.58	21.37	28.70	< 0.001
Middle-to-high intake		24.58	24.07	24.68	
Low-to-middle intake		24.41	24.22	24.44	
Low intake		23.43	30.34	22.18	
Dietary pattern 3 [cholesterols & PUFA] (%)					
High intake		23.27	14.70	24.82	< 0.001
Middle-to-high intake		24.72	23.55	24.93	
Low-to-middle intake		26.39	29.35	25.85	
Low intake		25.63	32.39	24.40	

*CKD* chronic kidney diseases, *SD* standard deviation, *CI* confidence interval, *BMI* Body mass index, *HDL* high density lipoprotein, *HOMA-IR* homeostatic model assessment of insulin resistance, *MUFA* monounsaturated fatty acids, *PUFA* polyunsaturated fatty acids

**Table 2:** Demographic characters and related risk factors of persons based on all stages of CKD

Characteristics		Overall Mean ± SD(%)	Stage 1	Stage 2	Stage 3a	Stage 3b	Stage 4	Stage 5	*p*-value
Sex	Male (%)	42.07	39.51	47.99	43.54	35.70	31.04	50.53	< 0.001
	Female (%)	57.93	60.49	52.01	56.46	64.30	68.96	49.47	
Age (Years), [mean (95% CI)]		60.0 (59.5–60.5)	42.1 (41.3–42.9)	63.3 (62.4–64.2)	69.3 (68.7–69.9)	73.9 (73.2–74.6)	72.5 (70.8–74.1)	60.2 (56.8–63.5)	< 0.001
Race/Ethnicity	Mexican American (%)	7.24	14.48	5.35	2.71	3.66	6.71	7.24	< 0.001
	Other Hispanic (%)	4.72	7.85	4.23	2.60	3.45	3.25	4.36	
	White (non-Hispanic) (%)	70.01	53.09	72.58	82.95	79.22	67.39	44.15	
	Non-Hispanic Black (%)	12.04	16.09	11.23	8.03	9.57	16.65	36.47	
	Other (%)	5.99	8.49	6.62	3.72	4.10	6.01	7.78	
BMI (kg/m^2^)		29.94 ± 0.11	30.04 ± 0.23	30.18 ± 0.25	29.56 ± 0.18	30.35 ± 0.34	30.46 ± 0.54	28.58 ± 0.84	0.128
Waist circumference (cm)		102.90 ± 0.27	100.81 ± 0.56	104.67 ± 0.58	103.19 ± 0.43	104.9 ± 0.72	105.45 ± 1.21	99.83 ± 2.16	< 0.001
Triglycerides (mg/dL)		141.50 ± 2.57	146.71 ± 5.7	147.03 ± 4.44	133.68 ± 3.25	142.59 ± 6.53	166.97 ± 12.23	115.35 ± 9.22	0.063
Total cholesterol (mg/dL)		192.60 ± 0.69	198.25 ± 1.28	192.86 ± 1.42	192.32 ± 1.23	181.91 ± 1.91	179.77 ± 3.31	181.17 ± 5.64	< 0.001
High density lipoprotein		52.92 ± 0.27	52.85 ± 0.55	52.99 ± 0.56	53.97 ± 0.45	50.79 ± 0.69	49.8 ± 1.23	52.65 ± 1.91	0.007
Serum triglycerides/HDL cholesterol ratio		3.28 ± 0.10	3.65 ± 0.23	3.27 ± 0.14	2.91 ± 0.11	3.26 ± 0.21	4.08 ± 0.39	2.41 ± 0.31	0.016
Apolipoprotein (B) (mg/dL)		92.20 ± 0.61	94.44 ± 1.07	94.1 ± 1.15	89.68 ± 1.07	89.15 ± 1.94	89.86 ± 2.64	79.88 ± 4.45	< 0.001
Systolic blood pressure (mmHg)		130.80 ± 0.33	125.82 ± 0.57	135.37 ± 0.73	131.09 ± 0.55	132.89 ± 1	137.13 ± 2.07	135.84 ± 3.57	< 0.001
Diastolic blood pressure (mmHg)		68.15 ± 0.23	72.82 ± 0.39	70.06 ± 0.51	65.72 ± 0.38	61.16 ± 0.68	58.67 ± 1.41	66.62 ± 1.75	< 0.001
Fasting blood glucose (mg/dL)		120.90 ± 1.12	121.08 ± 2.1	129.95 ± 2.68	115.65 ± 1.37	123.06 ± 3.16	126.82 ± 5.68	114.69 ± 6.27	< 0.001
Plasma Insulin (uU/mL)		15.68 ± 0.60	16.6 ± 1.08	16.56 ± 0.91	14.75 ± 0.68	19.67 ± 3.46	16.2 ± 2.29	6.41 ± 0.7	0.095
HOMA-IR		5.28 ± 0.26	5.55 ± 0.43	5.71 ± 0.41	4.98 ± 0.38	7.02 ± 1.43	5.37 ± 0.8	1.77 ± 0.23	0.152
HbA1c (%)		6.14 ± 0.02	6.1 ± 0.05	6.26 ± 0.05	6.03 ± 0.03	6.28 ± 0.05	6.37 ± 0.09	6.13 ± 0.17	< 0.001
2-h blood glucose (mg/dL)		138.6 ± 1.83	130.3 ± 3.4	147.4 ± 4.0	139.49 ± 2.7	149.75 ± 5.3	166.3 ± 11.9	160.3 ± 16.1	< 0.001
Hypertension (%)		58.07	35.48	61.99	65.34	82.60	86.23	84.28	< 0.001
Diabetes (%)		25.16	18.17	28.80	22.68	36.15	46.43	51.48	< 0.001
Metabolic syndrome (%)		39.14	31.25	42.06	38.60	51.69	61.60	34.81	< 0.001
Dietary pattern 1 [saturated fatty acids & MUFA] (%)									
High intake		27.68	31.55	27.12	25.89	25.17	18.98	28.16	< 0.001
Middle-to-high intake		26.26	26.98	26.32	26.55	26.36	20.84	11.75	
Low-to-middle intake		23.67	20.67	21.56	26.60	24.84	33.95	24.60	
Low intake		22.39	20.80	25.00	20.95	23.63	26.23	35.50	
Dietary pattern 2 [vitamins & minerals] (%)									
High intake		27.58	28.78	31.57	26.70	22.51	21.18	11.48	< 0.001
Middle-to-high intake		24.58	24.20	24.89	25.01	23.90	27.85	15.78	
Low-to-middle intake		24.41	26.37	20.35	25.24	25.38	21.71	20.31	
Low intake		23.43	20.65	23.18	23.05	28.21	29.27	52.43	
Dietary pattern 3 [cholesterols & PUFA] (%)									
High intake		23.27	27.54	25.18	21.85	15.57	11.45	15.32	< 0.001
Middle-to-high intake		24.72	27.24	20.98	25.26	24.51	20.42	23.07	
Low-to-middle intake		26.39	23.90	26.29	27.51	27.42	30.94	42.81	
Low intake		25.63	21.32	27.55	25.39	32.51	37.20	18.80	

*CKD* chronic kidney diseases, *SD* standard deviation, *CI* confidence interval, *BMI* Body mass index, *HDL* high density lipoprotein, *HOMA-IR* homeostatic model assessment of insulin resistance, *MUFA* monounsaturated fatty acids, *PUFA* polyunsaturated fatty acids

The intake quartiles of the 3 dietary patterns are described as follows. In pattern 1, the saturated fatty acid and MUFA intakes of participants with late-stage CKD were 24.13%, 24.08%, 26.70%, and 25.10%, for the high, middle-to-high, low-to-middle, and low intake groups, respectively. The saturated fatty acid and MUFA intakes of participants with early-stage CKD were 28.32%, 26.65%, 23.12%, and 21.90% for the high, middle-to-high, low-to-middle, and low intake groups, respectively. In pattern 2, 21.37% of the participants with late-stage CKD had high intakes of vitamins and minerals, and 30.34% had low intakes. For the participants with early-stage CKD, 28.70% had high intakes of vitamins and minerals, and 22.18% had low intakes. For pattern 3, the cholesterol and PUFA intakes of participants with late-stage CKD were 14.7%, 23.6%, 29.4%, and 32.4% for the high, middle-to-high, low-to-middle, and low intake groups, respectively. The cholesterol and PUFA intakes of participants with early-stage CKD were 24.82%, 24.93%, 25.85%, and 24.40% for the high, middle-to-high, low-to-middle, and low intake groups, respectively. The percentage of participants who had high intake of dietary pattern 1, dietary pattern 2, and dietary pattern 3 was lower among participants with late-stage CKD than that of participants with early-stage CKD.

After employing the logistic regression model to adjust the age, sex, and race for pattern 2 (model 1), the adjusted model revealed that the odds ratios (OR) of late-stage CKD patients in the low, low-to-middle, and middle-to-high intake groups of in pattern 2 (vitamins and minerals) were 1.66 (95% confidence interval (CI): 1.32–2.09), 1.34 (95% CI: 1.06–1.69), and 1.26 (95% CI: 0.99–1.59), respectively, with the high intake group as a reference (Table 3). After adjusting for BMI, triglyceride level, HDL level, diabetes, and hypertension, only the low intake group exhibited a significant difference with the high intake group. Participants with lowest intakes of vitamins and minerals had an OR of 1.74 (95% CI: 1.21–2.50) for late-stage CKD. In pattern 3 (cholesterol and PUFA), the OR of participants with late-stage CKD in the middle-to-high, low-to-middle, and low intake groups were 1.29 (95% CI: 1.01–1.66), 1.29 (95% CI: 1.01–1.64), and 1.40 (95% CI: 1.10–1.78), respectively, with the high intake group as a reference. After adjusting for other variables, participants with a low intake of cholesterol and PUFAs had an OR of 1.66 (95% CI: 1.13–2.43) for late-stage CKD. Those in the low-to-middle intake group had an OR of 1.61 (95% CI: 1.11–2.33) for late-stage CKD. Intakes in pattern 1 exhibited no significant associations with the CKD stages. A sensitivity analysis using the second 24-h recall measurement from the NHANES cohort showed that participants with lower intake of pattern 2 had a higher association for late-stage CKD compared with participants with higher intake of pattern 2 (Supplementary Table 2). However, the difference of pattern 3 among 4 intake groups was not significant.

**Table 3:** Adjusted logistic regression models for analyzing the association between quartile for dietary pattern and risk of the different stages of CKD

Dietary patterns	Model 1		Model 2		Model 3	
	OR	95%CI	OR	95%CI	OR	95%CI
Dietary pattern 1 [saturated fatty acids & MUFA]						
High intake	1.00		1.00		1.00	
Middle-to-high intake	0.97	0.76–1.23	0.78	0.55–1.13	0.76	0.52–1.10
Low-to-middle intake	1.15	0.91–1.45	1.03	0.72–1.46	0.95	0.67–1.36
Low intake	1.17	0.92–1.47	1.00	0.70–1.42	0.89	0.62–1.28
Dietary pattern 2 [vitamins & minerals]						
High intake	1.00		1.00		1.00	
Middle-to-high intake	1.26	0.99–1.59	1.31	0.91–1.90	1.27	0.87–1.85
Low-to-middle intake	1.34	1.06–1.69	1.41	0.98–2.03	1.40	0.96–2.04
Low intake	1.66	1.32–2.09	1.76	1.24–2.51	1.74	1.21–2.50
Dietary pattern 3 [cholesterols & PUFA]						
High intake	1.00		1.00		1.00	
Middle-to-high intake	1.29	1.01–1.66	1.44	0.99–2.10	1.38	0.94–2.02
Low-to-middle intake	1.29	1.01–1.64	1.58	1.10–2.28	1.61	1.11–2.33
Low intake	1.40	1.10–1.78	1.60	1.11–2.32	1.66	1.13–2.43

Model 1: adjusted Age, Sex, and Race; Model 2: adjusted Age, Sex, Race, Hypertension, Triglyceride, and High density lipoprotein; Model 3: adjusted Age, Sex, Race, Hypertension, Triglyceride, and High density lipoprotein, Diabetes, and Body mass index

*CKD* chronic kidney diseases, *OR* odds ratio, *CI* confidence interval, *MUFA* monounsaturated fatty acids, *PUFA* polyunsaturated fatty acids

Because age is a big and important confounder, we also performed an extra round of age-matching analysis by 1:2 propensity score matching using age (Supplementary Table 3). After propensity score matching, there were 797 participants in the late-stage CKD group and 1594 participants in the early-stage CKD group. The result showed a similar finding with the main result in the Table 3. Based on Model 3, we added smoking, exercise, and income for adjustment (Model 4) because they may be associated with CKD (Supplementary Table 4). The result was similar with the Model 3.

**Fig. 2:**
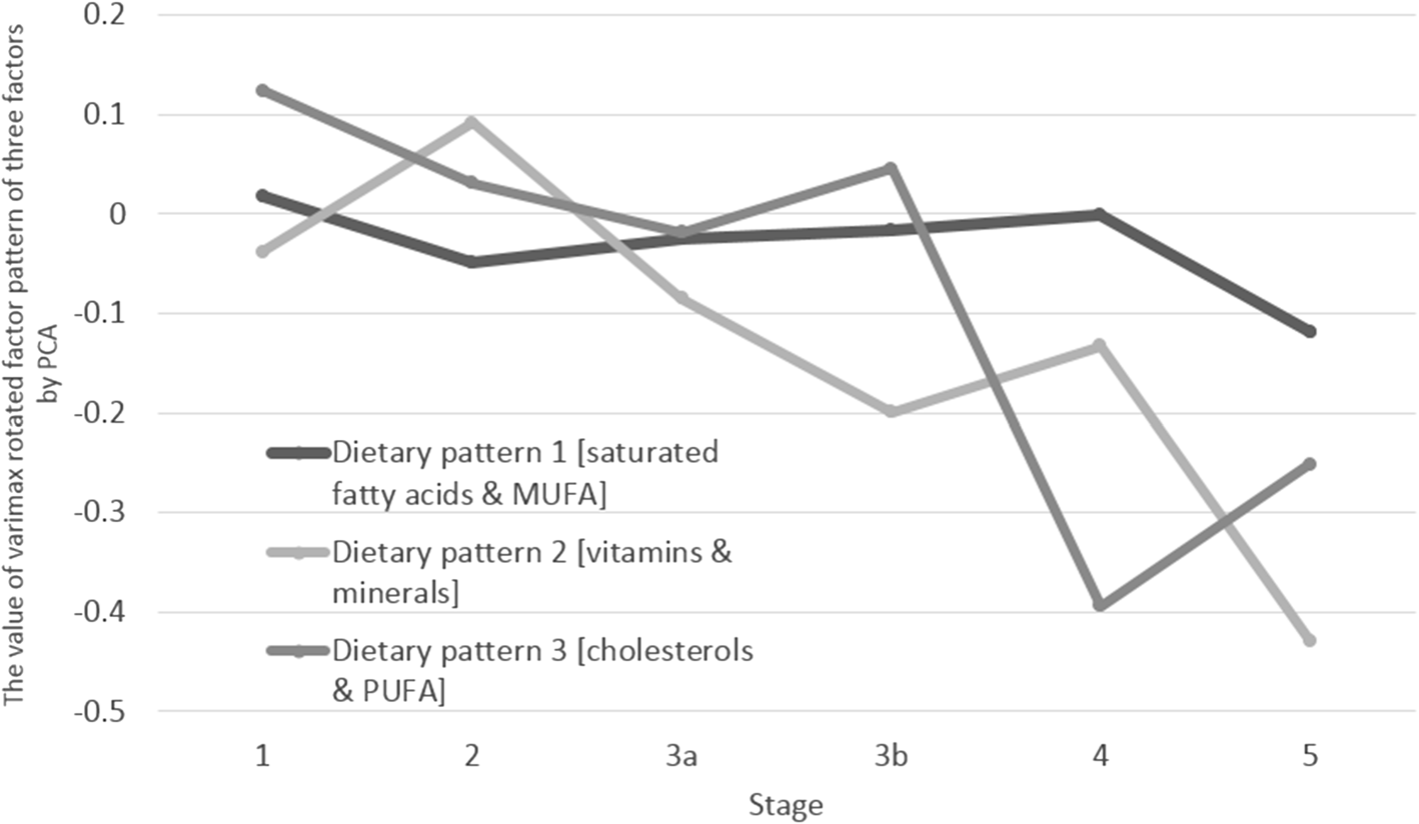
The value of varimax rotated factor pattern of three factors by PCA in different CKD stages. PCA, principal component analysis; CKD, chronic kidney disease

**Fig. 3:**
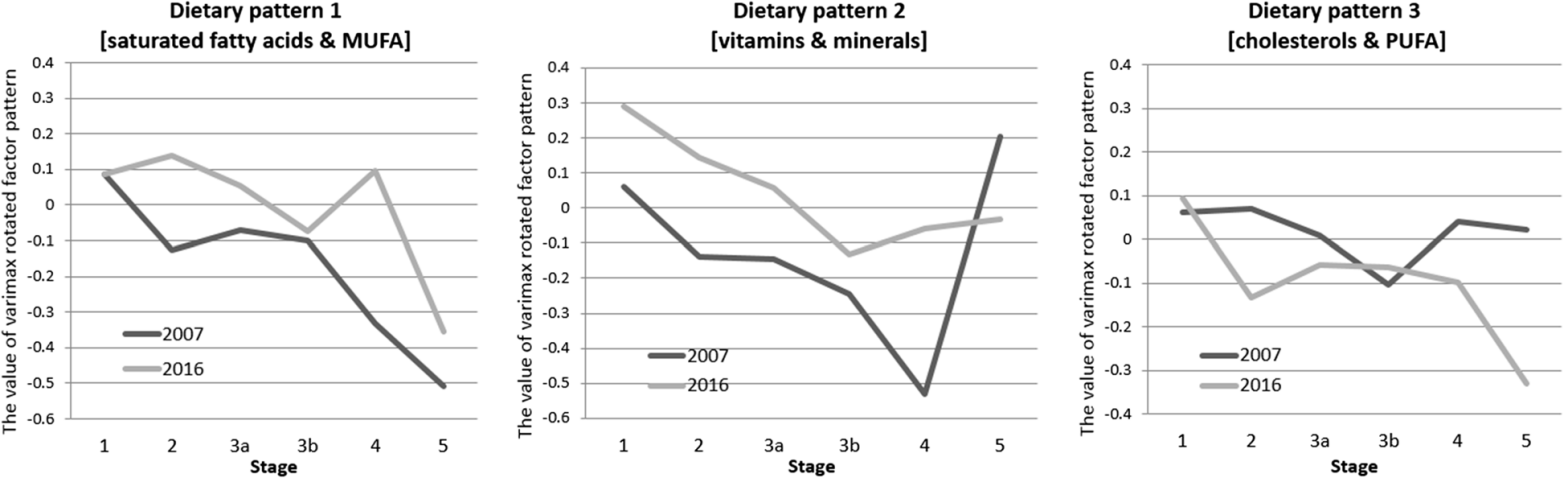
The changes of dietary patterns habits over time for any CKD stage, at distant time-points (2007 vs. 2016). CKD, chronic kidney disease

The value of varimax rotated factor pattern of three factors by PCA was presented to demonstrate the association between dietary patterns and CKD stages after adjusted all confounders. It indicated that higher intake of vitamins, minerals, cholesterol, and PUFAs was negatively associated with participants with late-stage CKD (Table 4 and Fig. 2). We compared the cross-sectional observations at distant time-points (2007 vs. 2016) and explored determinants of eventual changes (i.e., income and education) (Table 5). Compared with the data in 2007, intake of saturated fatty acids & MUFA and vitamins & minerals in 2016 was higher. Participants with higher income had higher intake of saturated fatty acids & MUFA, vitamins & minerals, and participants with higher education had higher intake of cholesterols & PUFA. The changes of dietary patterns habits over time for any CKD stage at distant time-points (2007 vs 2016) were also performed (Fig. 3).

**Table 4:** Adjusted general linear model for analyzing the association between each dietary pattern and CKD stages

	Dietary pattern 1 [saturated fatty acids and MUFA]	Dietary pattern 2 [vitamins and minerals]	Dietary pattern 3 [cholesterols and PUFA]
	Beta (95%CI)	Beta (95%CI)	Beta (95%CI)
Stage 1	Reference	Reference	Reference
Stage 2	-0.07 (-0.20–0.06)	0.13 (0.00–0.03)*	-0.09 (-0.23–0.05)
Stage 3a	-0.04 (-0.18–0.09)	-0.05 (-0.18–0.08)	-0.14 (-0.28–0.00)
Stage 3b	-0.03 (-0.20–0.14)	-0.16 (-0.33–0.00)	-0.08 (-0.26–0.10)
Stage 4	-0.02 (-0.25–0.21)	-0.09 (-0.32–0.13)	-0.52 (-0.77– -0.27)*
Stage 5	-0.14 (-0.44–0.17)	-0.39 (-0.69– -0.09)*	-0.38 (-0.70– -0.05)*

Adjusted by Age, Sex, Race, Hypertension, Triglyceride, and High density lipoprotein, Diabetes, and Body mass index

*CKD* chronic kidney diseases, *MUFA* monounsaturated fatty acids, *PUFA* polyunsaturated fatty acids, *CI* confidence interval

^*^P value < 0.05

**Table 5:** Adjusted general linear model for analyzing the association between each dietary pattern and CKD stages at distant time-points (2007 vs. 2016)

	Dietary pattern 1 [saturated fatty acids and MUFA]	Dietary pattern 2 [vitamins and minerals]	Dietary pattern 3 [cholesterols and PUFA]
**Stage**	Beta (95%CI)	Beta (95%CI)	Beta (95%CI)
Stage 1	Reference	Reference	Reference
Stage 2	-0.01 (-0.28–0.26)	0.00 (-0.29–0.29)	0.08 (-0.15–0.32)
Stage 3a	-0.10 (-0.38–0.19)	-0.20 (-0.51–0.10)	0.003 (-0.25–0.24)
Stage 3b	-0.09 (-0.45–0.27)	-0.17 (-0.57–0.22)	-0.10 (-0.42–0.22)
Stage 4	-0.09 (-0.54–0.36)	-0.36 (-0.85–0.13)	0.25 (-0.14–0.65)
Stage 5	0.23 (-0.58–1.04)	0.27 (-0.60–1.15)	0.02 (-0.68–0.72)
Year			
2007	Reference	Reference	Reference
2016	0.20 (0.03–0.38)*	0.36 (0.18–0.55)*	-0.01 (-0.17–0.14)
Income			
Below the poverty line	Reference	Reference	Reference
Above the poverty line	0.25 (0.07–0.38)*	0.27 (0.08–0.47)*	0.04 (-0.11–0.19)
Education			
Below High school	Reference	Reference	Reference
Above High school	0.66 (-0.33–1.64)	0.62 (-0.45–1.68)	1.03 (0.17–1.89)*

Adjusted by income, education age, Sex, Race, Hypertension, Triglyceride, and High density lipoprotein, Diabetes, and Body mass index

*CKD* chronic kidney diseases, *MUFA* monounsaturated fatty acids, *PUFA* polyunsaturated fatty acids, *CI* confidence interval

^*^P value < 0.05

To clarify the possibility of selection bias towards those who are particularly interested in some food, we excluded the participants who had weight loss or low-calorie diet, including low fat or cholesterol diet, low salt or sodium diet, sugar free or low sugar diet, low fiber diet, high fiber diet, diabetic diet, and another type of diet, for another analysis. The result showed a similar result with the Table 3 (Supplementary Table 5).

## Discussion

In this study, we analyzed the associations of the stages of CKD with dietary patterns among 4 480 participants in the NHANES database. The results indicated that among all 3 dietary patterns, the ratio of participants with high intake of nutrients was lower for participants with late-stage CKD; > 50% of participant with late-stage CKD were classified in the low-to-middle and low nutrient intake groups. Contrarily, the ratio of participants with high intakes of nutrients was higher among participants with early-stage CKD. Multivariate model analysis revealed that dietary patterns with lower intakes of vitamins and minerals and cholesterol and PUFAs were significantly associated with the stages of CKD. That is, participants with CKD who consumed lower levels of vitamins, minerals, cholesterols, and PUFAs in their daily diets were associated with late-stage CKD.

Mazidi et al. revealed that among patients who adopted the same dietary patterns as that in our study, the ratio of patients who had high nutrient intake was lower among patients with late-stage CKD [15]. In comparison, the ratio of patients who had high nutrient intake was higher among patients without CKD. In addition, the nutrient intake of these patients did not exhibit notable differences [15]. However, participants who were reported in the study as not having CKD consisted of those with normal kidney functions (i.e., the general population) and those with early-stage CKD. In comparison, the present study further verified that the nutrient intake of participants with late-stage CKD was relatively lower than that of participants with early-stage CKD.

Numerous studies have indicated that dietary micronutrients are associated with the occurrence and progression of CKD [5, 27, 28]. A nationwide study in the United States reported that higher intake of vitamins and minerals is negatively associated with CKD occurrence [15]. However, the associations between saturated fatty acids and MUFAs and CKD and between cholesterols & PUFA and CKD are not significant [15]. In addition, patients with CKD who were receiving dialysis exhibited risks of insufficient vitamin intake [14, 29, 30]. Asghari et al. suggested that higher intake of protein, carbohydrates, calcium, magnesium, potassium, and vitamin C reduced the occurrence of CKD by 43% [14]. For individuals without CKD, increased intake of magnesium effectively prevented CKD and mitigated the decline of kidney functions [30, 31]. Studies have also indicated that healthy dietary habits (such as the Mediterranean diet and DASH) and the intake of an abundance of antioxidant vitamins (e.g., vitamins E, C, and A), potassium, magnesium, calcium, dietary fiber, and Omega-3 fatty acids can reduce the risk of CKD occurrence [5, 12, 32–34]. The results were like those of the present study.

The progression and severity of CKD are closely associated to inflammation states [6]. The PUFAs in fats are anti-inflammatory and can improve patient health. Studies have demonstrated that higher intake of PUFA can reduce kidney damage among adults, maintain kidney function, and reduce the incidences of blood vessel complications [35–37]. As if also indicated that higher concentrations of PUFA, n-3 fatty acids, and n-6 fatty acid can mitigate the decline of kidney function [38]. In addition, PUFAs, such as eicosapentaenoic and docosahexaenoic acids, can attenuate the development of CKD [39]. Another study discussed the associations between nutrient intake and CKD incidence by using data from the Tehran Lipid and Glucose Study [40]. The results revealed that higher intakes of plant protein, PUFAs, and n-6 fatty acids was associated with lower CKD risk [40]. This was consistent with the results of this study, in which low intake of cholesterol and PUFAs were associated with late-stage CKD. In contrast to positive association between higher mortality and hypercholesteremia, previous study showed that higher cholesterol level was associated with lower mortality in the patients with chronic hemodialysis and low serum albumin levels [41]. Lower total cholesterol level is associated with increased mortality in dialysis patients due to cholesterol-lowering effect of systemic inflammation and malnutrition. Therefore, higher intake of cholesterol may be encouraged in the late-stage CKD who have systemic inflammation and malnutrition and a low albumin level [42].

Other studies have indicated that high-fat dietary patterns are directly associated with late-stage CKD risks [9]. Higher intakes of saturated fatty acids and lower intake of linoleic acids have been associated with metabolic syndrome [9, 14, 43]. However, in pattern 1 (saturated fatty acids and MUFAs) in this study, no significant difference was observed between participants with early and late-stage CKD. Insufficient intake of calories and protein can result in malnutrition [44]. This was possibly because all participants recruited into this study have CKD, who usually have strict dietary restrictions before the study. Therefore, the difference of pattern 1 (saturated fatty acids and MUFAs) between early-stage CKD and late-stage CKD may not be significant as the that between non-CKD and CKD. The fatty acids and protein in meat are important energy sources. In addition, some studies have indicated that the intake of protein from poultry and fish can slow the development of CKD [45]. Consumption of red meat is associated with higher risk of CKD [46]. However, red meat is also the optimal source of amino acids that are necessary for the human body. Therefore, for patients with CKD, adequate intake of meat may not increase the risk of CKD progression.

This study obtained sample data from the NHANES database, which serves as a representative data for the population of the United States. Consequently, the results of this study are applicable to the public in the United States. The NHANES database is cross-sectional, so causal relations could not be inferred. Some major limitations remain due to the nature of the study. The paper gives only a partial picture of the overall nutritional intakes because the analyzed patterns account for 50% of intakes and no information on macronutrients other than lipids are provided. Among macronutrients (source of energy), only lipids have been analyzed and since the model explaining only 50% of intakes, due to the lowering of lipid in advanced CKD, two alternative scenarios are possible: (1) unchanged or even reduced proteins and/or carbohydrates, in any case causing a reduction of energy intake; (2) increased proteins and/or carbohydrates to maintain an unchanged energy intake. What happens is unknown, but both these conditions may impact on CKD outcome. Although the demographic characteristics and related risk factors were controlled in the multivariate analysis, other unrecruited factors associated with CKD may also confounded the result. In addition, a 24-h review of the participants’ diet at specific time points was adopted to measure the participants’ dietary intakes. Thus, the dietary situations in the participants’ regular lives were not represented. During the research, participants’ dietary habits may change; therefore, the long-term dietary habits of the participants could not be inferred. However, this study only included participants that already had CKD, 28–59% of whom had received health education from professional doctors and followed specific dietary advice [47]. Consequently, the dietary habits during the interview period are likely to feature few changes, meaning that the study results were unlikely to have been influenced by dietary changes. The energy intake and nutritional status were not included into the analyses because the aim of the present study was to investigate the association between dietary patterns and different stages of CKD. We did not recruit the patients without CKD to compare with early-stage CKD and late-stage CKD, which would be substantially more interesting. Further studies about these issues are warranted in the future.

## Conclusions

For CKD participants, nutritional intake may be a crucial and modifiable risk factor for disease progression. In contrast to other studies, this study acquired sample data on participants with various stages of CKD and proposed risk and protection factors for early and late-stage CKD. This study indicated that higher intake of vitamins, minerals, cholesterol, and PUFAs was negatively associated with participants with late-stage CKD. Whether the results of this study can be used to effectively reduce the risks of disease progression among participants with CKD remains to be evaluated by subsequent clinical studies.

## Supplementary Information

**Additional file 1.**

**Additional file 2. **

**Additional file 3. **

**Additional file 4. **

**Additional file 5. **

**Additional file 6. **

## Data Availability

The NHANES used in the study is available publicly at: https://www.cdc.gov/nchs/nhanes/index.htm.

## Abbreviations

CKD: Chronic kidney disease
MUFA: Mono-unsaturated fatty acids
PUFA: Polyunsaturated fatty acids
CI: Confidence interval
NHANES: National Health and Nutrition Examination Survey
KDIGO: Kidney Disease Improving Global Outcomes
eGFR: Estimated Glomerular filtration rates
BMI: Body mass index
HDL: High-density lipoprotein
HOMA-IR: Homeostatic model assessment-insulin resistance
HbA1C: Glycated hemoglobin
FNDDS: Food and Nutrient Database for Dietary Studies
PCA: Principal component analysis
MEC: Mobile Examination Center
OR: Odds ratios
