# Supplementary material for: Associations between dietary patterns and stages of chronic kidney disease
Source: BMC Nephrol. 2022 Mar 22;23:115. doi: 10.1186/s12882-022-02739-1 (PMC8939097; doi:10.1186/s12882-022-02739-1)
Supplement: Supplementary file 1 — Additional file 1. [file 12882_2022_2739_MOESM1_ESM.docx]

**Supplementary Table 1.** Varimax rotated factor pattern of three factors

| dietary intake variables | Factor1 | Factor2 | Factor3 |
| --- | --- | --- | --- |
| SFA 14:0 (Tetradecanoic) (gm) | 0.93206 | 0.09262 | 0.05176 |
| SFA 10:0 (Decanoic) (gm) | 0.90673 | 0.09696 | -0.05789 |
| Total saturated fatty acids (gm) | 0.89353 | 0.12928 | 0.374650 |
| SFA 6:0 (Hexanoic) (gm) | 0.86198 | 0.09405 | -0.07825 |
| SFA 4:0 (Butanoic) (gm) | 0.85727 | 0.09714 | -0.07158 |
| SFA 8:0 (Octanoic) (gm) | 0.83167 | 0.07676 | -0.09027 |
| SFA 16:0 (Hexadecanoic) (gm) | 0.81518 | 0.13816 | 0.47771 |
| SFA 18:0 (Octadecanoic) (gm) | 0.80783 | 0.11915 | 0.43676 |
| Total fat (gm) | 0.71569 | 0.18069 | 0.60776 |
| Calcium (mg) | 0.60649 | 0.46599 | 0.09786 |
| SFA 12:0 (Dodecanoic) (gm) | 0.57399 | 0.02422 | -0.06539 |
| Phosphorus (mg) | 0.57153 | 0.49410 | 0.50311 |
| MFA 16:1 (Hexadecenoic) (gm) | 0.56001 | 0.04391 | 0.55441 |
| Total sugars (gm) | 0.42617 | 0.28860 | 0.08418 |
| Total Folate (mcg) | 0.25239 | 0.83722 | 0.15168 |
| Folate, DFE (mcg) | 0.23774 | 0.83055 | 0.07439 |
| Iron (mg) | 0.31412 | 0.75819 | 0.17618 |
| Folic acid (mcg) | 0.18227 | 0.72048 | -0.0873 |
| Thiamin (Vitamin B1) (mg) | 0.35093 | 0.70935 | 0.24940 |
| Vitamin B6 (mg) | 0.11738 | 0.66901 | 0.23711 |
| Riboflavin (Vitamin B2) (mg) | 0.46248 | 0.65701 | 0.16614 |
| Niacin (mg) | 0.21822 | 0.64380 | 0.44283 |
| Magnesium (mg) | 0.34987 | 0.62655 | 0.40693 |
| Added vitamin B12 (mcg) | -0.02062 | 0.60726 | -0.19844 |
| Potassium (mg) | 0.38487 | 0.59373 | 0.42191 |
| Dietary fiber (gm) | 0.22537 | 0.59137 | 0.24206 |
| Vitamin A, RAE (mcg) | 0.17653 | 0.56400 | 0.02089 |
| Zinc (mg) | 0.40806 | 0.55858 | 0.30587 |
| Food folate (mcg) | 0.21209 | 0.55753 | 0.37663 |
| Vitamin B12 (mcg) | 0.12879 | 0.52456 | 0.13476 |
| Copper (mg) | 0.15918 | 0.52171 | 0.21759 |
| Vitamin E as alpha-tocopherol (mg) | 0.21668 | 0.52036 | 0.37565 |
| Carbohydrate (gm) | 0.4876 | 0.49524 | 0.27112 |
| Retinol (mcg) | 0.26457 | 0.48108 | -0.05263 |
| Added alpha-tocopherol (Vitamin E) (mg) | -0.04327 | 0.43289 | -0.14597 |
| Vitamin C (mg) | -0.00238 | 0.41711 | 0.06236 |
| PFA 20:4 (Eicosatetraenoic) (gm) | 0.19244 | 0.05818 | 0.72026 |
| MFA 20:1 (Eicosenoic) (gm) | 0.20036 | 0.09845 | 0.68217 |
| Total polyunsaturated fatty acids (gm) | 0.39339 | 0.19591 | 0.67815 |
| Total choline (mg) | 0.34174 | 0.31742 | 0.67684 |
| Protein (gm) | 0.43749 | 0.41501 | 0.65738 |
| PFA 18:2 (Octadecadienoic) (gm) | 0.39686 | 0.19177 | 0.65578 |
| Total monounsaturated fatty acids (gm) | 0.62081 | 0.16537 | 0.63809 |
| Selenium (mcg) | 0.34397 | 0.39095 | 0.63293 |
| MFA 18:1 (Octadecenoic) (gm) | 0.61032 | 0.16728 | 0.62968 |
| Cholesterol (mg) | 0.37435 | 0.06900 | 0.62286 |
| PFA 22:5 (Docosapentaenoic) (gm) | -0.10045 | 0.04785 | 0.61974 |
| PFA 22:6 (Docosahexaenoic) (gm) | -0.21051 | 0.04428 | 0.59088 |
| Sodium (mg) | 0.44989 | 0.36305 | 0.57921 |
| PFA 18:3 (Octadecatrienoic) (gm) | 0.37846 | 0.19293 | 0.56998 |
| PFA 20:5 (Eicosapentaenoic) (gm) | -0.21082 | 0.06259 | 0.52377 |
| Theobromine (mg) | 0.24596 | 0.05138 | 0.01008 |
| Moisture (gm) | 0.23372 | 0.29870 | 0.25235 |
| Caffeine (mg) | 0.17972 | 0.08645 | 0.05602 |
| Lycopene (mcg) | 0.08006 | 0.23272 | 0.06854 |
| Alcohol (gm) | 0.00871 | 0.06898 | 0.17760 |
| PFA 18:4 (Octadecatetraenoic) (gm) | -0.02929 | 0.02995 | 0.36329 |
| Alpha-carotene (mcg) | -0.03354 | 0.19920 | 0.03614 |
| Vitamin K (mcg) | -0.04317 | 0.26558 | 0.26543 |
| Beta-cryptoxanthin (mcg) | -0.04643 | 0.13843 | 0.03192 |
| MFA 22:1 (Docosenoic) (gm) | -0.09069 | 0.02065 | 0.35650 |
| Lutein + zeaxanthin (mcg) | -0.09248 | 0.2368 | 0.18852 |
| Beta-carotene (mcg) | -0.09403 | 0.30129 | 0.13319 |
