# Supplementary material for: Associations between dietary patterns and stages of chronic kidney disease
Source: BMC Nephrol. 2022 Mar 22;23:115. doi: 10.1186/s12882-022-02739-1 (PMC8939097; doi:10.1186/s12882-022-02739-1)
Supplement: Supplementary file 2 — Additional file 2. [file 12882_2022_2739_MOESM2_ESM.docx]

**Supplementary Table 2.** Adjusted logistic regression models for analyzing the association between quartile for dietary pattern and risk of the different stages of CKD using the second 24-hour recall measurement from the NHANES cohort

| Dietary patterns | Model 1 | | Model 2 | | Model 3 | |
| --- | --- | --- | --- | --- | --- | --- |
|  | OR | 95%CI | OR | 95%CI | OR | 95%CI |
| Dietary pattern 1 [saturated fatty acids & MUFA] | | | | | | |
| High intake | 1.00 |  | 1.00 |  | 1.00 |  |
| Middle-to-high intake | 0.90 | 0.71–1.16 | 0.67 | 0.46–0.99 | 0.65 | 0.44–0.97 |
| Low-to-middle intake | 1.09 | 0.86–1.39 | 1.05 | 0.73–1.52 | 1.00 | 0.69–1.46 |
| Low intake | 1.05 | 0.83–1.34 | 0.94 | 0.66–1.35 | 0.85 | 0.59–1.23 |
| Dietary pattern 2 [vitamins & minerals] | | | | | | |
| High intake | 1.00 |  | 1.00 |  | 1.00 |  |
| Middle-to-high intake | 1.25 | 0.97–1.60 | 1.55 | 1.05–2.28 | 1.51 | 1.01–2.27 |
| Low-to-middle intake | 1.53 | 1.20–1.96 | 1.96 | 1.34–2.87 | 1.95 | 1.31–2.90 |
| Low intake | 1.60 | 1.25–2.04 | 1.94 | 1.32–2.86 | 1.93 | 1.30–2.88 |
| Dietary pattern 3 [cholesterols & PUFA] | | | | | | |
| High intake | 1.00 |  | 1.00 |  | 1.00 |  |
| Middle-to-high intake | 1.02 | 0.80–1.31 | 1.29 | 0.88–1.88 | 1.26 | 0.85–1.86 |
| Low-to-middle intake | 0.94 | 0.73–1.20 | 0.93 | 0.63–1.38 | 0.94 | 0.63–1.41 |
| Low intake | 1.08 | 0.85–1.38 | 1.21 | 0.83–1.76 | 1.25 | 0.85–1.83 |

Model 1: adjusted Age, Sex, and Race; Model 2: adjusted Age, Sex, Race, Hypertension, Triglyceride, and High density lipoprotein; Model 3: adjusted Age, Sex, Race, Hypertension, Triglyceride, and High density lipoprotein, Diabetes, and Body mass index.

Abbreviations: CKD, chronic kidney diseases; NHANES; National Health and Nutrition Examination Survey, OR, odds ratio; CI, confidence interval; MUFA, monounsaturated fatty acids; PUFA, polyunsaturated fatty acids.
