# Supplementary material for: Associations between dietary patterns and stages of chronic kidney disease
Source: BMC Nephrol. 2022 Mar 22;23:115. doi: 10.1186/s12882-022-02739-1 (PMC8939097; doi:10.1186/s12882-022-02739-1)
Supplement: Supplementary file 3 — Additional file 3. [file 12882_2022_2739_MOESM3_ESM.docx]

**Supplementary Table 3.** Adjusted logistic regression models for analyzing the association between quartile for dietary pattern and risk of the different stages of CKD after age-matching analysis by 1:2 propensity score matching using age

| Dietary patterns | Model 1 | | Model 2 | | Model 3 | |
| --- | --- | --- | --- | --- | --- | --- |
|  | OR | 95%CI | OR | 95%CI | OR | 95%CI |
| Dietary pattern 1 [saturated fatty acids & MUFA] (%) | | | | | | |
| High intake | 1.00 |  | 1.00 |  | 1.00 |  |
| Middle-to-high intake | 0.91 | 0.71–1.18 | 0.79 | 0.54–1.16 | 0.79 | 0.53–1.17 |
| Low-to-middle intake | 1.17 | 0.91–1.50 | 1.16 | 0.80–1.69 | 1.08 | 0.74–1.59 |
| Low intake | 1.23 | 0.96–1.57 | 1.18 | 0.81–1.71 | 1.05 | 0.71–1.54 |
| Dietary pattern 2 [vitamins & minerals] (%) | | | | | | |
| High intake | 1.00 |  | 1.00 |  | 1.00 |  |
| Middle-to-high intake | 1.29 | 1.01–1.66 | 1.35 | 0.92–1.99 | 1.33 | 0.89–1.98 |
| Low-to-middle intake | 1.36 | 1.06–1.69 | 1.44 | 0.98–2.12 | 1.49 | 1.00–2.21 |
| Low intake | 1.61 | 1.26–2.05 | 1.68 | 1.16–2.45 | 1.69 | 1.15–2.49 |
| Dietary pattern 3 [cholesterols & PUFA] (%) | | | | | | |
| High intake | 1.00 |  | 1.00 |  | 1.00 |  |
| Middle-to-high intake | 1.33 | 1.03–1.73 | 1.49 | 1.00–2.22 | 1.44 | 0.95–2.16 |
| Low-to-middle intake | 1.28 | 0.99–1.66 | 1.56 | 1.06–2.29 | 1.57 | 1.06–2.33 |
| Low intake | 1.43 | 1.10–1.84 | 1.60 | 1.09–2.37 | 1.69 | 1.12–2.53 |

Model 1: adjusted Sex, and Race; Model 2: adjusted Sex, Race, Hypertension, Triglyceride, and High density lipoprotein; Model 3: adjusted Sex, Race, Hypertension, Triglyceride, and High density lipoprotein, Diabetes, and Body mass index.
