# Supplementary material for: Associations between dietary patterns and stages of chronic kidney disease
Source: BMC Nephrol. 2022 Mar 22;23:115. doi: 10.1186/s12882-022-02739-1 (PMC8939097; doi:10.1186/s12882-022-02739-1)
Supplement: Supplementary file 4 — Additional file 4. [file 12882_2022_2739_MOESM4_ESM.docx]

**Supplementary Table 4.** Logistic regression models for analyzing the association between quartile for dietary pattern and risk of the different stages of CKD after adjustment of age, sex, race, hypertension, triglyceride, high density lipoprotein, diabetes, body mass index, smoking, exercise, and income

| Dietary patterns | Model 4. Adjusted age, sex, race, hypertension, triglyceride, high density lipoprotein, diabetes, body mass index, smoking, exercise, and income | |
| --- | --- | --- |
|  | OR | 95%CI |
| Dietary pattern 1 [saturated fatty acids & MUFA] (%) | | |
| High intake | 1.00 |  |
| Middle-to-high intake | 0.97 | 0.76–1.23 |
| Low-to-middle intake | 1.15 | 0.91–1.45 |
| Low intake | 1.17 | 0.92–1.47 |
| Dietary pattern 2 [vitamins & minerals] (%) | | |
| High intake | 1.00 |  |
| Middle-to-high intake | 1.26 | 0.99–1.59 |
| Low-to-middle intake | 1.34 | 1.06–1.69 |
| Low intake | 1.66 | 1.32–2.09 |
| Dietary pattern 3 [cholesterols & PUFA] (%) | | |
| High intake | 1.00 |  |
| Middle-to-high intake | 1.29 | 1.01–1.66 |
| Low-to-middle intake | 1.29 | 1.01–1.64 |
| Low intake | 1.40 | 1.10–1.78 |

Abbreviations: CKD, chronic kidney diseases; OR, odds ratio; CI, confidence interval; MUFA, monounsaturated fatty acids; PUFA, polyunsaturated fatty acids.
