# Supplementary material for: Associations between dietary patterns and stages of chronic kidney disease
Source: BMC Nephrol. 2022 Mar 22;23:115. doi: 10.1186/s12882-022-02739-1 (PMC8939097; doi:10.1186/s12882-022-02739-1)
Supplement: Supplementary file 5 — Additional file 5. [file 12882_2022_2739_MOESM5_ESM.docx]

**Supplementary Table 5.** Adjusted logistic regression models for analyzing the association between quartile for dietary pattern and risk of the different stages of CKD after excluding the participants who had weight loss or low-calorie diet, including low fat or cholesterol diet, low salt or sodium diet, sugar free or low sugar diet, low fiber diet, high fiber diet, diabetic diet, and another type of diet (n=3609)

| Dietary patterns | Model 1 | | Model 2 | | Model 3 | |
| --- | --- | --- | --- | --- | --- | --- |
|  | OR | 95%CI | OR | 95%CI | OR | 95%CI |
| Dietary pattern 1 [saturated fatty acids & MUFA] (%) | | | | | | |
| High intake | 1.00 |  | 1.00 |  | 1.00 |  |
| Middle-to-high intake | 1.04 | 0.79–1.36 | 0.83 | 0.54–1.36 | 0.83 | 0.53–1.28 |
| Low-to-middle intake | 1.15 | 0.88–1.51 | 1.01 | 0.67–1.53 | 0.98 | 0.65–1.49 |
| Low intake | 1.04 | 0.79–1.36 | 0.89 | 0.58–1.36 | 0.83 | 0.56–1.32 |
| Dietary pattern 2 [vitamins & minerals] (%) | | | | | | |
| High intake | 1.00 |  | 1.00 |  | 1.00 |  |
| Middle-to-high intake | 1.12 | 0.84–1.48 | 1.17 | 0.76–1.82 | 1.15 | 0.73–1.81 |
| Low-to-middle intake | 1.25 | 0.95–1.65 | 1.23 | 0.80–1.90 | 1.28 | 0.82–1.99 |
| Low intake | 1.52 | 1.16–1.98 | 1.51 | 1.01–2.29 | 1.52 | 0.99–2.33 |
| Dietary pattern 3 [cholesterols & PUFA] (%) | | | | | | |
| High intake | 1.00 |  | 1.00 |  | 1.00 |  |
| Middle-to-high intake | 1.44 | 1.08–1.92 | 1.63 | 1.03–2.59 | 1.53 | 0.96–2.45 |
| Low-to-middle intake | 1.14 | 0.85–1.52 | 1.60 | 1.01–2.52 | 1.56 | 0.99–2.48 |
| Low intake | 1.49 | 1.18–1.98 | 2.01 | 1.28–3.59 | 2.03 | 1.28–3.22 |

Model 1: adjusted Age, Sex, and Race; Model 2: adjusted Age, Sex, Race, Hypertension, Triglyceride, and High density lipoprotein; Model 3: adjusted Age, Sex, Race, Hypertension, Triglyceride, and High density lipoprotein, Diabetes, and Body mass index.

Abbreviations: CKD, chronic kidney diseases; OR, odds ratio; CI, confidence interval; MUFA, monounsaturated fatty acids; PUFA, polyunsaturated fatty acids.
