# Supplementary figures and images for: Associations between dietary patterns and stages of chronic kidney disease
Source: BMC Nephrol. 2022 Mar 22;23:115. doi: 10.1186/s12882-022-02739-1 (PMC8939097; doi:10.1186/s12882-022-02739-1)

**Supplementary Figure 1.** The turning point of the dietary pattern in the scree test

**
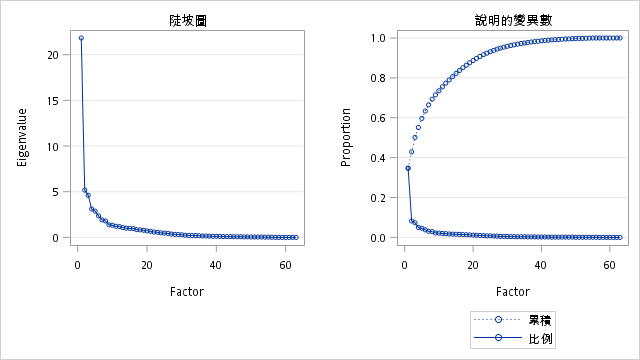
**

Supplement: Supplementary file 6 — Additional file 6. [file 12882_2022_2739_MOESM6_ESM.docx]
